# Effect of Chemical Composition of Metal–Organic Crosslinker on the Properties of Fracturing Fluid in High-Temperature Reservoir

**DOI:** 10.3390/molecules29122798

**Published:** 2024-06-12

**Authors:** Shenglong Shi, Jinsheng Sun, Shanbo Mu, Kaihe Lv, Jingping Liu, Yingrui Bai, Jintang Wang, Xianbin Huang, Jiafeng Jin, Jian Li

**Affiliations:** 1 College of Science, Qingdao University of Technology, Qingdao 266520, China; 2 Department of Petroleum Engineering, China University of Petroleum (East China), Qingdao 266580, Chinawangjintang@upc.edu.cn (J.W.); 20170092@upc.edu.cn (X.H.); cuplijian@sina.com (J.L.); 3 CNPC Engineering Technology R&D Company Limited, Beijing 102206, China; 4 Shandong Three Carbon Technology Development Co., Ltd., Dongying 257100, China

**Keywords:** chemical composition, aluminum–zirconium crosslinker, gelation mechanism, fracturing fluid, high temperature

## Abstract

To investigate the effect of the chemical composition of a metal–organic crosslinker on the performances of fracturing fluid in high-temperature conditions, four zirconium (Zr) crosslinkers and one aluminum–zirconium (Al-Zr) crosslinker with a polyacrylamide were used. The crosslinkers possessed the same Zr concentration, but they differed in component amounts and the order of the addition of the crosslinker components, leading to different chemical compositions in the crosslinkers. The fracturing fluids prepared by different tested crosslinkers were compared in terms of properties of rheological behavior, sand-carrying ability, microstructure, and gel breaking characteristics. The results showed that the fracturing fluids prepared by zirconium lactic acid, ethanediamine, and sorbitol crosslinkers offered the slowest viscosity development and highest final viscosity compared to the zirconium lactic acid crosslinker and the zirconium lactic acid and ethanediamine crosslinker. The zirconium sorbitol, lactic acid, and ethanediamine crosslinker exhibited a faster crosslinking rate and a higher final viscosity than the zirconium lactic acid, ethanediamine, and sorbitol crosslinker; the crosslinker showed crosslinking density and crosslinking reactivity, resulting in more crosslinking sites and a higher strength in the fracturing fluid. The Al-Zr-based crosslinker possessed better properties in temperature and shear resistance, viscoelasticity, shear recovery, and sand-carrying ability than the Zr-based crosslinker due to the synergistic crosslinking effect of aluminum and zirconium ions. The tertiary release gelation mechanism of the Al-Zr-based fracturing fluid achieved a temperature resistance performance in the form of continuous crosslinking, avoiding the excessive crosslinking dehydration and reducing viscosity loss caused by early shear damage. These results indicated that the chemical compositions of metal–organic crosslinkers were important factors in determining the properties of fracturing fluids. Therefore, the appropriate type of crosslinker could save costs without adding the additional components required for high-temperature reservoirs.

## 1. Introduction

Hydraulic fracturing technology is a vital tool in the exploration and development of unconventional oil and gas resources [1,2,3]. During the fracturing process, fracturing fluid is pumped into formation under the action of high-pressure pumps; this allows the horizontal and vertical fractures in the formation to be widened, as well as communicating with tiny transverse natural fractures and carrying large quantities of proppants into the fractures. After the fracturing fluid is broken, the proppants support the formation and provide a stable support channel for the flow of hydrocarbons. Therefore, a fracturing fluid with excellent sand-carrying and delayed crosslinking properties enables significant increases in oil and gas production [4,5,6].

Polymers are the primary components of fracturing fluids, but higher polymer concentrations may lead to more gel residue after the gel breaking of a fracturing fluid. In addition, higher polymer concentrations will result in lower fracture conductivity and productivity [7,8]. Therefore, the complete breaking of a fracking fluid is important, and a breaker is required to clean up the fracture after the fracturing construction. Nevertheless, under the conditions of high temperature and high salinity, the residual gel can damage the fracture channel if the polymer is not completely broken up; this is because of the uneven distribution of the breaker and the high polymer amount. Therefore, a formulation of fracturing fluid with low polymer loading is advantageous [9,10,11].

Adding the appropriate amount of crosslinker to a polymer solution will improve the performance of the fracturing fluid and reduce the concentration of the polymer used, reducing formation damage and costs [12,13,14,15,16]. At present, common crosslinkers used in hydraulic fracturing are boron (B) crosslinker, aluminum (Al) crosslinker, and zirconium (Zr) crosslinker in hydraulic fracturing [17,18]. Although B crosslinkers have the ability to crosslink effectively with guar and their fracturing fluid possesses good shear resistance, they have limited high-temperature resistance, and their coordination bond with guar gum will break at 140 °C [19,20]. In comparison, Zr crosslinkers are a good choice for high-temperature conditions; however, the disadvantage of fracturing fluids formed with commercial zirconium crosslinkers is poor shear resistance [21,22,23]. Therefore, it is necessary to use matched components to control the release rate of metal ions from the crosslinkers, thereby delaying the crosslinking of the polymer and crosslinker until the fracturing fluid passes through the high-shear environment [24,25]. In addition, the component type of a Zr crosslinker can determine the final rheological properties when the other components of the crosslinker are the same. Sokhanvarian et al. investigated the effect of three different component types (lactate, lactate–propylene glycol, and lactate–triethanol amine) on the performance of fracturing fluids formed by these three Zr crosslinkers; they showed that the fracturing fluid formed by a Zr–triethanol amine–lactate crosslinker and polyacrylamide had the best performance at 90–200 °C; the reactivity of the Zr crosslinker in solution was found to be influenced by its chemical composition [26].

In addition to the introduction of components in the crosslinker, adding another crosslinker to the zirconium crosslinker is also a way to achieve a wider application and address the shortcomings of the zirconium crosslinker [27,28]. Dual crosslinkers (boron–zirconium crosslinker or aluminum–zirconium crosslinker) have been successfully developed and applied in the field [29]. Nasr-El-Din et al. used three Zr crosslinkers, one B-Zr crosslinker, and one Al-Zr crosslinker to form carboxymethyl hydroxypropyl guar-crosslinked polymers. The Al-Zr crosslinker was able to maintain a higher viscosity at high shear rates and exhibited better shear recovery as compared to the B-Zr crosslinker and the Zr crosslinker [30]. Yang et al. prepared an organic B-Zr crosslinker through tertiary release crosslinking strategy, and the components were glycerol, sorbitol, and citric acid. When the temperature was raised above 60 °C, boron ions were gradually released, which enhanced the strength of the gel and thus improved the sand-carrying capacity. When the temperature reached 140 °C, the complexed zirconium ions began to work, and the fracturing fluid was suitable for temperatures up to 180 °C; the fracturing fluid could be completely broken, leaving little residue after the operation [31]. Nevertheless, to the best of our knowledge, the effects of different components and different metal combinations on the performance of Zr-based crosslinked fracturing fluids in high-temperature and high-salinity conditions have not been discussed in depth.

Therefore, zirconium ion (Zr^4+^) and aluminum ion (Al^3+^) with different component amounts were employed as metal–organic crosslinkers, and an anionic polyacrylamide polymer was as the thickener. The objectives of the present study are as follows: (1) to investigate the effect of the chemical composition of metal–organic crosslinkers on the temperature and shear resistance of crosslinked polymers with four zirconium crosslinkers; (2) to systematically compare the temperature- and shear-resistance ability, shear recovery performance, viscoelasticity, sand-carrying performance, and gel-breaking capacity of an Al-Zr crosslinker and a Zr crosslinker; (3) to use scanning electron microscopy (SEM) to uncover the gelation mechanism of fracturing fluid. Thus, this study provides technical support and a reference for further explorations into the chemical compositions of metal–organic crosslinkers for fracturing fluids.

## 2. Results and Discussion

### 2.1. Temperature- and Shear-Resistance Performance

During the process of pumping fracturing fluids into the formation, the fracturing fluids were subjected to shear, high-temperature and high-salt environments; these adversely affect the rheological properties of fracturing fluids. Therefore, it was necessary to assess the temperature- and shear-resistance performance of fracturing fluid in order to simulate the conditions that a fracturing fluid might encounter. The effects of component amounts, the order of addition of the crosslinker components, the metal type of the crosslinkers, and the polymer type on the temperature- and shear-resistance performances of the fracturing fluids were investigated.

#### 2.1.1. Influence of Component Amount of Crosslinker

**Figure 1:**
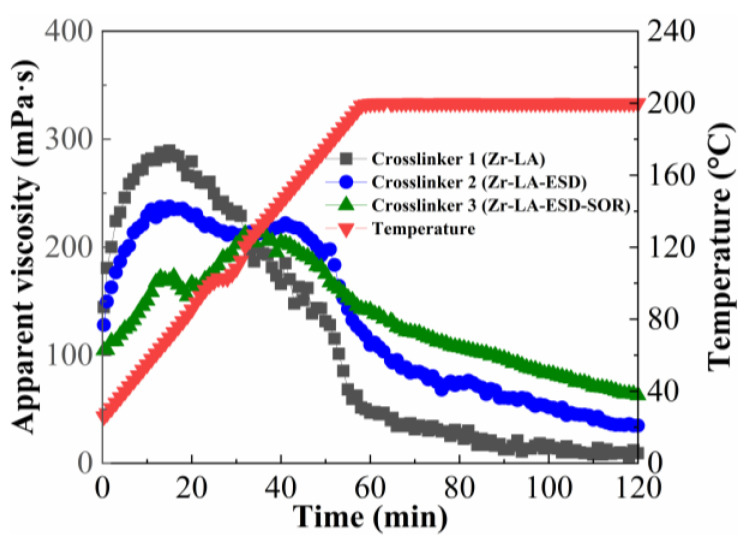
Influence of component amount of crosslinker on temperature- and shear-resistance performance of fracturing fluid.

The fracturing fluid was prepared with 1.2 wt% ASC and 0.15 wt% crosslinker; ASC was a kind of polymer thickener, which was synthesized by acrylamide, acrylic acid, 2-acrylamide-2-methylpropanesulfonic acid, and acryloyl morpholine. Figure 1 showed the viscosity of ASC crosslinked with crosslinker 1 (zirconium lactate—Zr-LA), crosslinker 2 (zirconium lactic acid and ethanediamine—Zr-LA-ESD), and crosslinker 3 (zirconium lactic acid, ethanediamine, and sorbitol—Zr-LA-ESD-SOR) at 200 °C, respectively; this showed the influence of the component amounts of the crosslinkers on the temperature- and shear-resistance performances of the fracturing fluids. It could be observed that all three zirconium crosslinkers produced sufficient thermal and rheological stability, which suggested that they are able to successfully carry the proppant (viscosity ≥ 100 mPa·s) during the initial stage of temperature increase. Zr-LA had a faster crosslinking rate compared with the other two crosslinkers; the viscosity of the fracturing fluid showed the change trend of growing first, reaching a peak, and then rapidly decreasing. Zr-LA-ESD exhibited a medium speed reaction and a medium final viscosity of the fracturing fluid; meanwhile, Zr-LA-ESD-SOR resulted in the slowest generation of viscosity and the highest final viscosity under these conditions.

It can be seen from Figure 1 that different component types for the crosslinkers had a remarkable effect on the viscosity change of the fracturing fluids at a high temperature. The lower the number of components, the faster the rate of increase in the viscosity of the fracturing fluid system, and the higher the peak viscosity, the lower the final viscosity was. For crosslinker Zr-LA, zirconium ions were crosslinked with the carboxylate group on the thickener chain at low temperatures; the faster crosslinking rate resulted in an unstable viscosity peak at the beginning of the crosslinking period. The viscosity continued to decrease as the test continued, suggesting that crosslinking was relatively ineffective when the components amount of the crosslinker is lower. However, with the increase in the components amount of the crosslinker, the crosslinking time of the fracturing fluid was prolonged, and its viscosity could still be maintained at a high level after shearing for 120 min; this shows better temperature- and shear-resistance performance. The components amount in the crosslinker increased, with stronger chelation strength in the components with zirconium ions and higher temperatures required for the release of the zirconium ions; this resulted in a slower release rate of the zirconium ions and a lower crosslinking rate between the crosslinking agent and the polymer [32]. This excellent temperature- and shear-resistance ability stemmed from the higher crosslinking density, resulting from an increase in the components amount in the crosslinker, which could be achieved by generating more crosslinked networks along the polymer skeleton. This contributed to reducing the shear damage of the fracturing fluid in the early stage and allowed for higher viscosity at high temperatures in the later stages of crosslinking [33]. Fracturing fluids formed by the crosslinker Zr-LA-ESD-SOR had the slowest increase in viscosity and the highest final viscosity because they possessed the maximum number of components and had the strongest components attached to the zirconium ions.

#### 2.1.2. Influence of Order of Addition of Crosslinker Components

**Figure 2:**
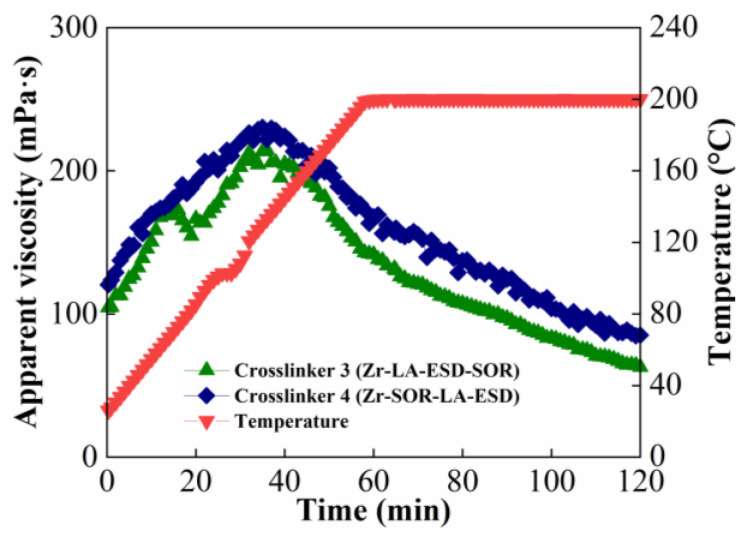
Influence of order of addition of crosslinker components on temperature- and shear-resistance performance of fracturing fluid.

In order to examine the influence of the order of the addition of the crosslinker components on the temperature- and shear-resistance performance of fracturing fluids, crosslinker 3 (Zr-LA-ESD-SOR) and crosslinker 4 (zirconium sorbitol, lactic acid, and ethanediamine—Zr-SOR-LA-ESD) were selected; the concentrations of the crosslinker and the thickener were 0.15 wt% and 1.2 wt%, respectively. The results are shown in Figure 2.

The viscosity of both of the fracturing fluids increased rapidly and then gradually decreased after 20 min of shearing, which might be a result of the unhindered movement and dispersion of the crosslinker in the polymer solution. However, due to the delayed crosslinking characteristics of the crosslinkers, both of the systems were not fully crosslinked and weak gels were formed. The fracturing fluid’s viscosity then increased once more and quickly dropped when the temperature rose above 140 °C, signifying that the crosslinking sites had been liberated and the macromolecular chains had fully crosslinked. This ultimately caused the system’s elasticity to increase until equilibrium was attained. The findings demonstrated that, even though the crosslinkers had the same kind and amounts of components, the zirconium crosslinkers’ various component addition orders gave them distinct characteristics. In comparison to crosslinker Zr-LA-ESD-SOR, crosslinker Zr-SOR-LA-ESD demonstrated a faster crosslinking rate and a higher final viscosity, suggesting that Zr-SOR-LA-ESD had a higher level of reactivity. The final equilibrium viscosity rose as crosslinker reactivity increased because more crosslinking sites meant greater strength [34].

Therefore, when the crosslinking agent was synthesized, the component with a strong chelating ability was preferred for addition, and the component with a weak chelating ability was added later. As the temperature increases, the component with a weak chelating ability was located in the outermost layer of the crosslinker, and it was decided that we should release the crosslinker for the first stage of crosslinking. When the temperature increased further, the component with strong chelating ability released the crosslinker to form the second level of crosslinking. This continued to achieve the purpose of multi-level release. The multistage, controlled release of the delayed crosslinker allowed for the gradual crosslinking of the fracturing fluid and reduced the increased friction of the fracturing fluid caused by the sharp increase in viscosity.

#### 2.1.3. Influence of Metal Type of Crosslinker

**Figure 3:**
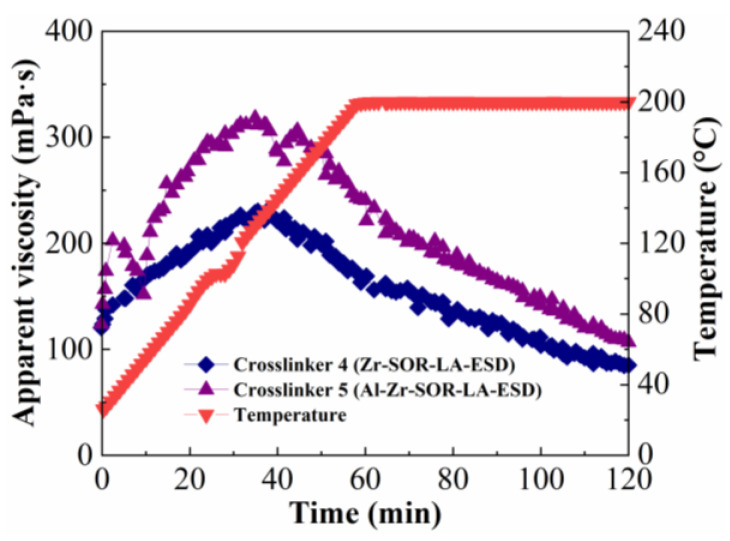
Influence of metal type of crosslinker on temperature- and shear-resistance performance of fracturing fluid.

In order to examine the influence of metal type of crosslinker on the temperature- and shear-resistance performance of fracturing fluid, crosslinker 4 (Zr-SOR-LA-ESD) and crosslinker 5 (aluminum zirconium sorbitol, lactic acid, and ethanediamine—Al-Zr-SOR-LA-ESD) were chosen; the concentrations of the crosslinker and the thickener were 0.15 wt% and 1.2 wt%, respectively. The results are shown in Figure 3.

As shown in Figure 3, two kinds of fracturing fluids were heated in the temperature range of 25–200 °C after constant shear at 170 s^−1^ for 2 h. For the ASC crosslinked with the Al-Zr crosslinker, the viscosity eventually remained above 100 mPa·s, indicating that the fracturing fluid had good temperature and shear resistance. However, for the ASC crosslinked with the Zr crosslinker, the value of final viscosity was only 68 mPa·s under the same conditions. Compared with Zr-based fracturing fluid, the viscosity of the Al-Zr-based fracturing fluid increased significantly and displayed better temperature and shear resistance, which might be related to the tertiary release process of the crosslinker. The viscosity of the Al-Zr-based fracturing fluid firstly increased above 200 mPa·s after shearing 5 min and then decreased to 150 mPa·s; this might be related to the crosslinking of the aluminum ion and the pre-shear damage that was caused when the temperature was below 50 °C. This further reacted with carboxyl groups from ASC to produce the first physical crosslinked network, thus increasing the viscosity of the solution. Subsequently, as temperatures continued to rise, the carboxyl groups of the ASC crosslinked with primary complexed zirconium ion in situ form a second crosslinked network under the high-temperature condition; thus, the viscosity increased to more than 300 mPa·s, and it began to decrease immediately after the temperature increased to over 140 °C. However, there was only a brief drop in viscosity, followed by a small increase; this might be due to the crosslinking of secondary complexed zirconium ion to generate third crosslinked network. This excellent thermal stability resulted from a higher crosslinking density from tertiary crosslinking, which created more crosslinks along the backbone [29,30,35]. Therefore, the unusual thermal stability characteristics of the Al-Zr-based fracturing fluid are desirable in maintaining fracture width and ensuring proppant transport inside reservoir fractures at ultra-high temperatures.

#### 2.1.4. Influence of Polymer Type

**Figure 4:**
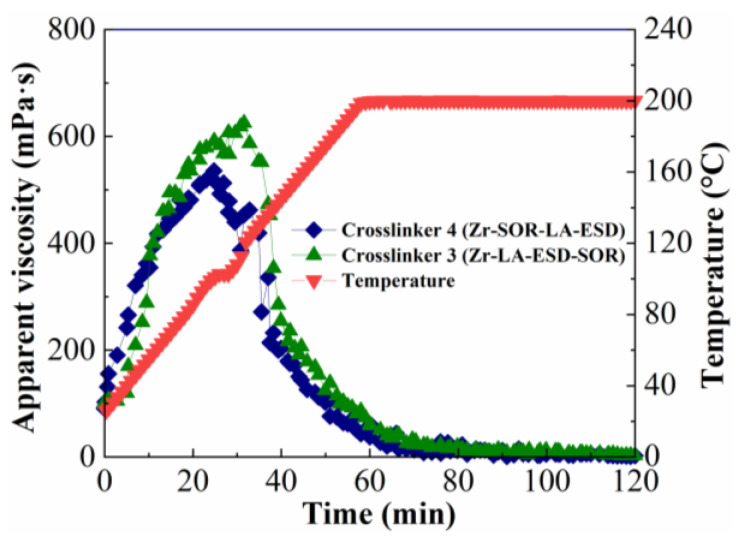
Influence of the type of polymer on the temperature- and shear-resistance performance of fracturing fluid.

To determine the influence of the type of polymer on the temperature- and shear-resistance performance of fracturing fluid, CMHPG solutions were prepared at the same conditions as the ASC solution; crosslinker 3 (Zr-La-ESD-Sor) and crosslinker 4 (Zr-Sor-La-ESD) were selected, and the concentrations of the crosslinker and the polymer were 0.30 wt% and 0.40 wt%, respectively. The results are in Figure 4.

It was found that CMHPG crosslinked with crosslinker 3 outperformed crosslinker 4 at temperatures ranging from 25 to 200 °C. This finding is in opposition to the ASC experiments, which demonstrated that crosslinker 4 provided superior performance. This discrepancy in performance was caused by the low reactivity of crosslinker 4, which prevented it from producing any appreciable viscosity with the CMHPG during the experiments. The two polymers’ varying shear and temperature tolerances were the cause of the variations in the behavior of the individual crosslinkers with polyacrylamide and CMHPG. The hydrodynamic radius of the polymer ASC in solution was altered when a specific quantity of AMPS monomer was introduced to the polyacrylamide structure. This resulted in more hydrogen bonding sites, increased stiffness, and increased charge density, as well as a mixture of hydrophobic and hydrophilic segments. Morpholine rings in ACMO were weakly sensitive to salt brine and had electric neutrality. They also allowed the polymer’s main chain to become more rigid and less flexible, as well as to expand its curling angle in brine conditions. These elements might improve the polymer’s essential qualities in solution, including salt tolerance, shear resistance, and thermal stability.

### 2.2. Shear Recovery

**Figure 5:**
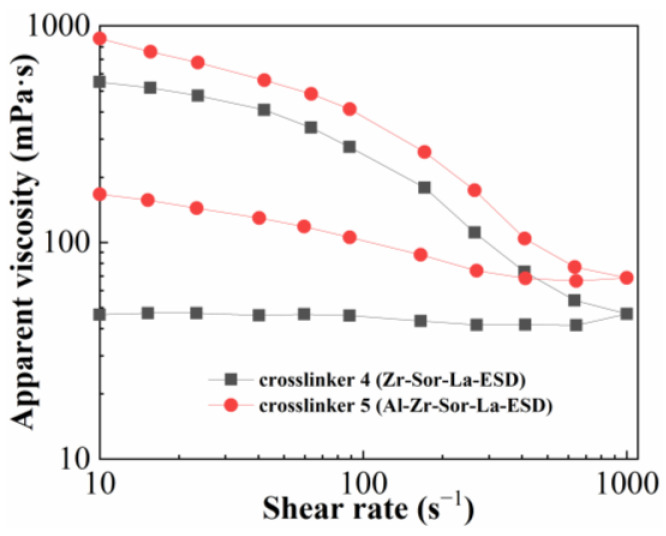
Influence of crosslinker type on shear recovery performance of fracturing fluid.

Crosslinker 4 (Zr-SOR-LA-ESD) and crosslinker 5 (Al-Zr-SOR-LA-ESD) were chosen to examine the influence of crosslinker type on the shear recovery performance of fracturing fluid; the concentrations of crosslinker and thickener were 0.15 wt% and 1.2 wt%, respectively. The results are shown in Figure 5.

The irreversible nature of Zr-based crosslinker was proven when the ASC crosslinked with crosslinker 4 was tested under a shear ramp of 10 to 1000 s^−1^ and subsequently dropped from 1000 to 10 s^−1^; the viscosity of the fracturing fluid was not entirely recovered. The viscosity loss following shear was considerably lower with an Al-Zr-based crosslinker (crosslinker 5) than with a Zr-based crosslinker (crosslinker 4) when the ASC reverted from a high shear rate to a low shear rate (according to the same shear sweep test). Because more metal ions in the crosslinker produced more crosslinking sites, the network strength of the fracturing fluid grew as the quantity of metal ions in the crosslinker increased. This suggested that more metal ions in the crosslinker would increase the final strength of the crosslinked system [28,36]. Because it could maintain its viscosity even after being exposed to high shear rates, the Al-Zr-based crosslinker used in this study proved to be a better option for fracturing applications than Zr-based crosslinkers.

### 2.3. Viscoelasticity

**Figure 6:**
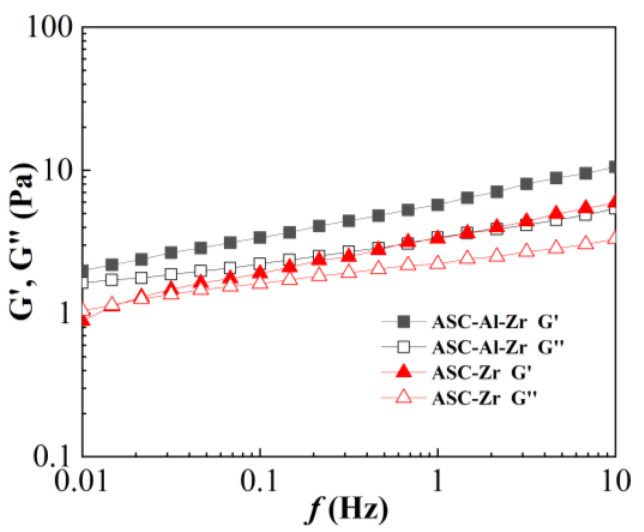
Viscoelastic modulus curve of the fracturing fluid produced by Zr-based crosslinker and Al-Zr-based crosslinker.

The HAAKE rheometer was used to set up thirty measurement points, which were then scanned at 0.01–10 Hz to examine the relationship between shear force, the loss modulus G″, and the storage modulus, G′. The experimental results are displayed in Figure 6. This was used to describe the viscoelastic properties of the fracturing fluid produced by crosslinkers 4 (Zr-SOR-LA-ESD) and 5 (Al-Zr-SOR-LA-ESD). The concentrations of crosslinker and thickener were 0.15 wt% and 1.2 wt%, respectively.

The sample based on ASC-Zr exhibited a characteristic viscoelastic response, meaning that the initial loss modulus of the fracturing fluid was greater, and its storage modulus was lower. Nonetheless, the storage modulus grew quickly as the frequency increased and tended to cross over with the loss modulus; this suggested that, at high shear rates, the elasticity of gel might eventually surpass its viscosity. Figure 6 illustrated that the storage modulus and loss modulus of crosslinked fracturing fluid gels based on ASC-Al-Zr did not intersect; both the storage modulus and the loss modulus of the ASC-Al-Zr system were higher than those of the ASC-Zr system. A high storage modulus is typically an indicator of a fracturing fluid’s superior sand-carrying performance. The crosslinking of the Al-Zr-based crosslinker could enable the fracturing fluid to have a good viscoelasticity and an excellent sand-carrying capacity; this is indicated by the storage modulus and the loss modulus being almost parallel in the 0.01 to 10 Hz frequency range. This further confirmed that the crosslinking action of aluminum ions and zirconium ions could work synergistically to improve the viscoelasticity of the fracturing fluid [30,31].

### 2.4. Sand-Carrying Performance

Transporting the proppant along the length of the fracture and maintaining its suspension were the main tasks performed by the fracturing fluid. Previous analyses of fracturing fluids based on Zr and Al-Zr had shown that they could sustain high viscosities at high temperatures. It was anticipated that these two types of fracturing fluid would be able to suspend sand. Using 20–40 mesh ceramic particles suspended in these two types of fracturing fluid with 1.2 wt% ASC and 0.15 wt% crosslinker at 90 °C, proppant sedimentation tests were conducted to examine the sand-carrying capabilities of two fracturing fluids made with crosslinker 4 (Zr-SOR-LA-ESD) and crosslinker 5 (Al-Zr-SOR-LA-ESD).

**Figure 7:**
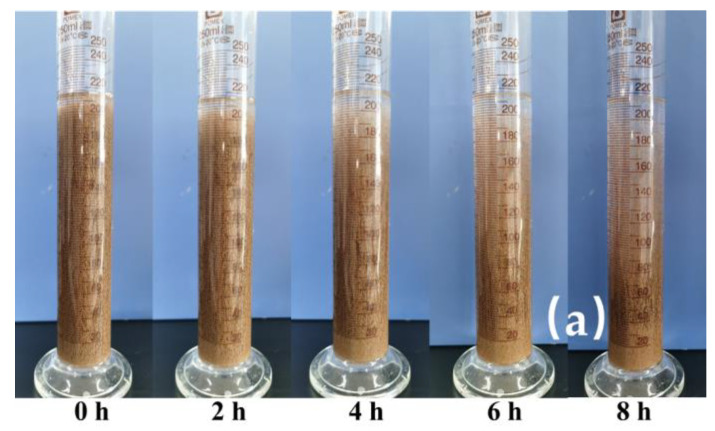

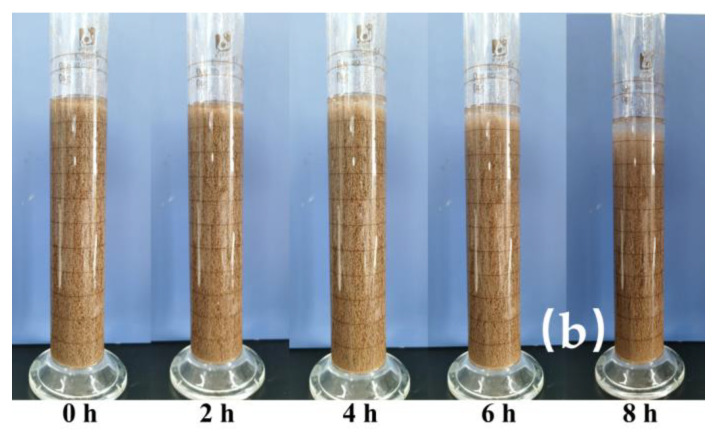
The proppant carrying capacity of the fracturing fluid produced by Zr-based crosslinker and Al-Zr-based crosslinker. (**a**) Zr-based crosslinker. (**b**) Al-Zr-based crosslinker.

Figure 7 showed that the change trends in the state of the suspended proppant were almost the same for both crosslinkers. As the observation time increased, the proppant in the fracturing fluid settled gradually and a clear liquid began to appear in the upper layer of the fracturing fluid. However, the two crosslinkers had different degrees of proppant settlement. The settling velocity of ceramic particles with Al-Zr-based fracturing fluid was slower than that of Zr-based fracturing fluid. This might be due to the higher viscous nature and denser crosslinked network of Al-Zr-based fracturing fluid compared to the Zr-based fracturing fluid, aluminum ions combined with zirconium ions crosslinked with ASC to strengthen the viscoelasticity of the system. As a result, the Al-Zr-based fracturing fluid suspended more than 90% of the proppants and achieved good sand-carrying and deep-sand-spreading characteristics [37]. Consequently, the Al-Zr-based fracturing fluid possessed significant sand-suspending properties at high temperatures and offered great potential for fracturing processes in harsh environments.

### 2.5. Microstructure of the Fracturing Fluid

**Figure 8:**
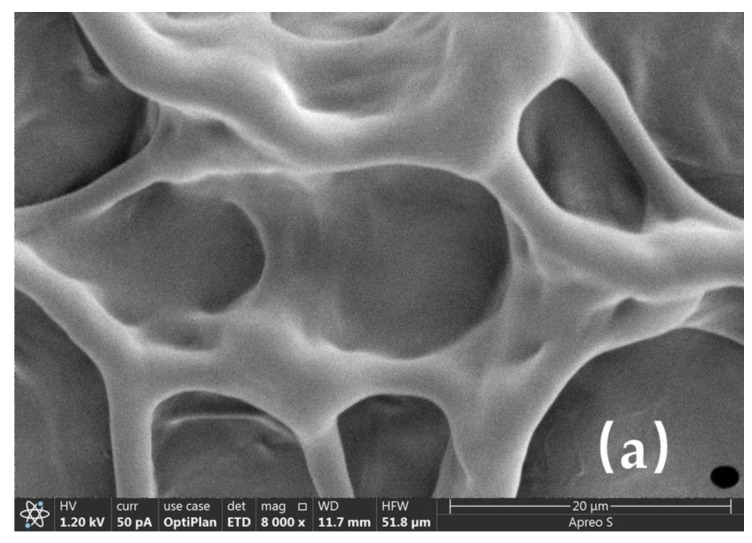

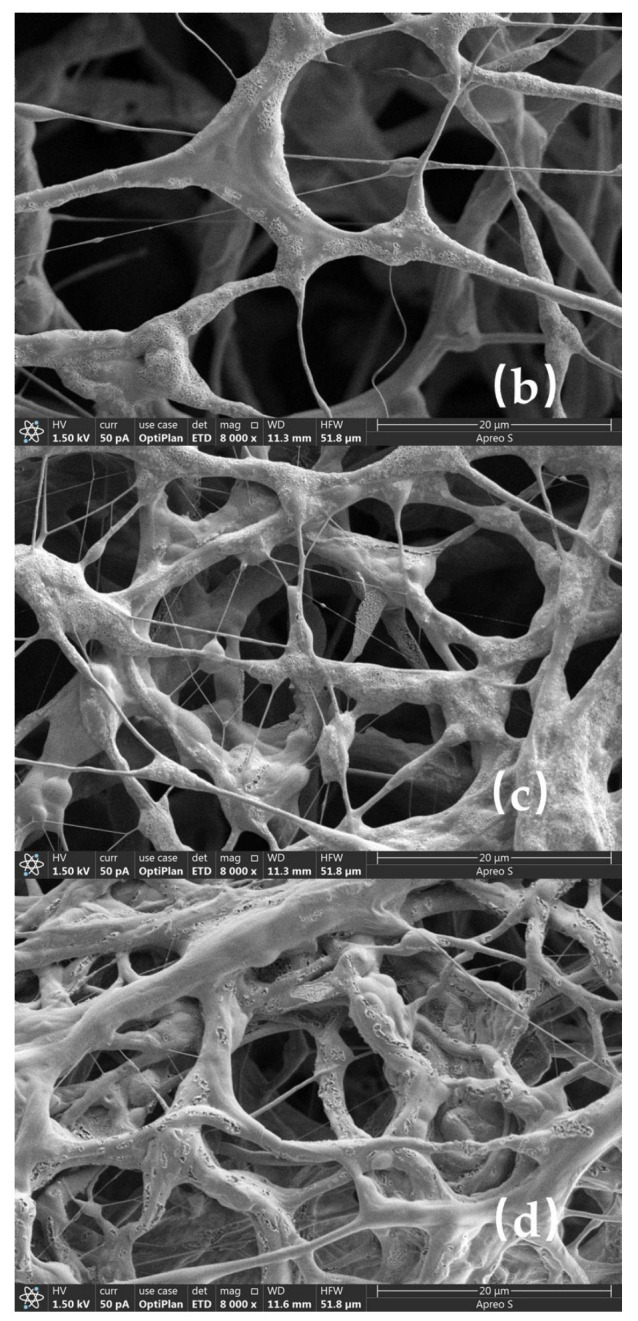
Micrographs of the fracturing fluid prepared with different crosslinkers and ASC. (**a**) ASC. (**b**) ASC–crosslinker 2 (Zr-LA-ESD). (**c**) ASC–crosslinker 4 (Zr-SOR-LA-ESD). (**d**) ASC–crosslinker 5 (Al-Zr-SOR-LA-ESD).

The morphology of the fracturing fluid was observed using a scanning electron microscope (SEM) to examine its microstructure. The fracturing fluid was generated using 1.2% ASC and 0.15% crosslinker. The outcomes are displayed in Figure 8.

Figure 8a shows an SEM picture of the ASC solution; the polymer’s network structure was comparatively loose. ASC–crosslinker 2 (Zr-LA-ESD) had a three-dimensional network structure and secondary networks formed in conjunction with the polymer skeletons as a result of physical crosslink production (Figure 8b), which raised the viscosity. Because of the more stable crosslinking in the ASC–crosslinker 4 (Zr-SOR-LA-ESD) system, the gel skeleton seemed to have a prominent multilayered three-dimensional structure, and the subnetworks became tighter and tighter, which increased the viscosity even more (Figure 8c). Furthermore, as Figure 8d illustrates, the ASC–crosslinker 5 (Al-Zr-SOR-LA-ESD) system created a more intricate network by crosslinking zirconium ions and aluminum ions with ASC, ultimately resulting in a comparatively high viscosity. The ASC–crosslinker 5 system performed better at extremely high temperatures than the single crosslinking systems did, as a result of the subnetwork’s development, which strengthened the network structure. Furthermore, as illustrated in Figure 8a–d, the samples exhibited a more liquid-like behavior. In contrast, the ASC–crosslinker 5 system with a tertiary crosslinked network enabled a moderate flow to the bottle cap when the sample bottle was turned upside down. When combined, these findings confirmed that the enhanced thermal stability was caused by the synergistic crosslinking action of aluminum ions and zirconium ions, which was in line with the rheological findings.

### 2.6. Gel Breaking

At the final stage of the hydraulic fracturing process, the high-viscosity fluid typically needs to be drained out of the reservoir. Therefore, it is imperative that the fracturing fluid be broken quickly and completely prior to the subsequent flowback. The gel-breaking test was conducted at 200 °C using Zr-based and Al-Zr-based fracturing fluids. The findings are shown in Table 1. Table 1 demonstrates that both the fracturing fluids’ final viscosities reached less than 5 mPa·s in less than 4 h; the gel breaking time was within 4 h, which meets the condition for delayed gel breaking in the absence of capsule wrapping. The broken gel liquids were translucent and yellowish, and the residual contents in both systems were less than 200 mg/L, which complied with the standards. This further illustrated the benefits of using an Al-Zr-based fracturing fluid in large-displacement, low-permeability, unconventional reservoirs due to its low reservoir damage and its ability to prevent the blockage of flow paths [38].

### 2.7. Gelation Mechanism of Al-Zr-Based Fracturing Fluid

**Figure 9:**
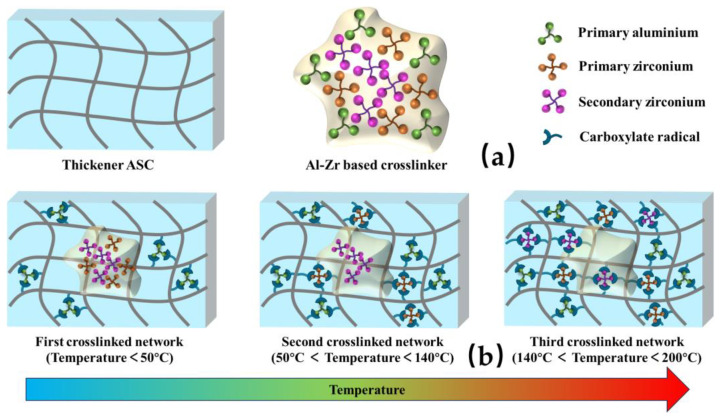
Gelation mechanism diagram of Al-Zr-based fracturing fluid. (**a**) Diagram of thickener and crosslinker. (**b**) Diagram of gelation mechanism diagram.

Studies had indicated that, during the pumping process, fracturing fluids experience varied levels of shear, which might result in different degrees of damage to the gel structures. Shear damage in the formation fracture was negligible, while the most significant damage occurred in the perforation, the ground pipeline, and the wellbore. The multistage aluminum zirconium composite crosslinker system was created to maintain the viscosity of the fracturing fluid under high-reservoir-temperature circumstances following high-speed shearing; the gelation mechanism is shown in Figure 9.

The main complexed aluminum ions were progressively released to create weak crosslink at temperatures below 50 °C; this process often occurred in surface pipelines and wellbore. The main complexed zirconium ions were progressively released as the wellbore temperature increased above 50 °C, strengthening the gel’s ability to hold the proppants. Relatively weak crosslinking connections created by the main zirconium ion helped to prevent shear damage at the wellbore and perforation. Nevertheless, shearing quickly disrupted the crosslinking links, particularly at temperatures exceeding 140 °C. By now, the secondary complexed zirconium crosslinker had begun to function, and the zirconium ion had created considerably stronger crosslinking links. The gel that had been crosslinked by the zirconium ions had demonstrated good temperature tolerance as a result of the strong crosslinking bonds [31]. The combined effects of zirconium ions and aluminum ions resulted in a notable improvement in the thermal stability of the Al-Zr-based fracturing fluid. Thus, at extremely high temperatures, the unique thermal stability feature of this Al-Zr-based fracturing fluid was found to be perfect for preserving fracture breadth and guaranteeing proppant transit into reservoir cracks.

## 3. Materials and Methods

### 3.1. Materials

**Figure 10:**
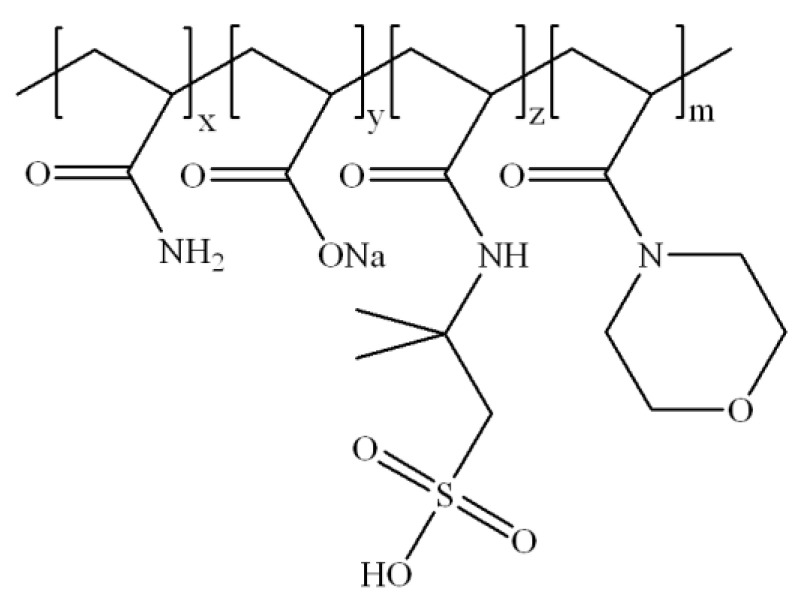
The structural formula of ASC.

**Figure 11:**
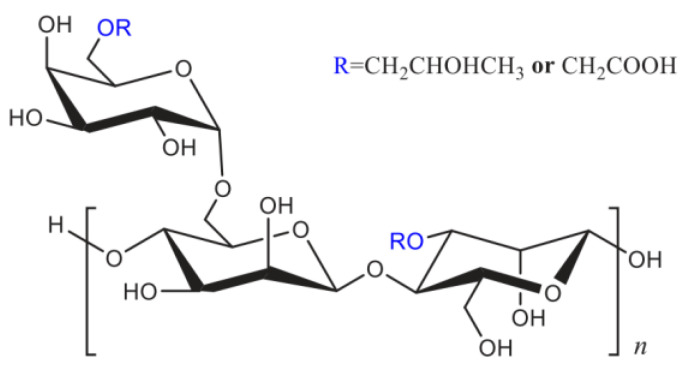
The structural formula of CMHPG.

Zirconium oxychloride (ZrOCl_2_·8H_2_O, AR 98%), aluminum chloride (AlCl_3_, AR 98%), lactic acid (LA, AR 98%), ethanediamine (ESD, AR 98%), sorbitol (SOR, AR 98%), sodium chloride (NaCl, AR 99.5%), calcium chloride (CaCl_2_, AR 98%), sodium hydroxide (NaOH, AR 98%), and ammonium persulfate (APS, AR 98%) were purchased from Shanghai Aladdin BioChem Technology Co., Ltd. (Shanghai, China) The thickener (a copolymer of AM, AA, AMPS and ACMO, denoted as ASC) was synthesized in the laboratory, as depicted in Figure 10 [39]. Thickener 2 is a biopolymer; CMHPG (industrial grade, molecular weight of 1.36 × 10^6^ g/mol) polymer powder is made by Shandong AK Biotech Co., Ltd., (Dongying, China) and its chemical composition is presented in Figure 11. Proppant ceramsite with an apparent density of 2.45 g/cm^3^ and a size of 40/70 mesh was supplied by Jingang New Materials Co., Ltd. (Zouping, China). Deionized (DI) water was obtained from a water purification system. Compound salt brine prepared with sodium chloride and calcium chloride at a mass ratio of 15:1 was synthesized in the lab, which was simulated formation water with a total dissolved solids of 30,000 mg/L in Shengli oilfield in China. All chemicals were utilized without further purification.

### 3.2. Synthesis of Metal–Organic Crosslinker

The temperature of the constant temperature oil bath pot was set to 60 °C; certain amounts of the metal compound and the distilled water were added to the beaker; the mixture was stirred for 10 min under a nitrogen atmosphere. Afterwards, different components were added successively to the beaker; after the first component was added, the mixture was stirred with a glass rod for 30 min before the second component was added; the second component was stirred with the mixture for 30 min before the third component was added, and so on. The mixture was stirred until the liquid became faint yellow and transparent. Then, the pH value of the mixture solution was adjusted to 7 by adding NaOH solution. Finally, the mixture was stirred continuously for 5 h to obtain a metal–organic crosslinker. The compositions of the crosslinkers were given in Table 1. The crosslinker 1 is zirconium lactate (the abbreviation is Zr-LA), crosslinker 2 is zirconium lactate and ethanediamine (the abbreviation is Zr-LA-ESD), crosslinker 3 is zirconium lactate, ethanediamine, and sorbitol (the abbreviation is Zr-LA-ESD-SOR), crosslinker 4 is zirconium sorbitol and lactate and ethanediamine (the abbreviation is Zr-SOR-LA-ESD), and crosslinker 5 is aluminum zirconium sorbitol and lactate and ethanediamine (the abbreviation is Al-Zr-SOR-LA-ESD). Crosslinker 3 and crosslinker 4 in Table 2 were used to evaluate the influence of order of addition of crosslinker components on performance of fracturing fluid; crosslinker 3 Zr-LA-ESD-SOR and crosslinker 4 Zr-SOR-LA-ESD were chosen.

### 3.3. Preparation of Fracturing Fluid

The specified amount of ASC was gradually added to the compound salt brine under continuous mechanical stirring for 15 min. Then, a certain amount of crosslinker was added to the above polymer solution, and the gel was stirred thoroughly with a glass rod to acquire a pickable gel.

### 3.4. Temperature- and Shear-Resistance Performance

Using the HAKKE MARS 40 rheometer (Thermo Fisher Scientific, Waltham, MA, USA), the temperature- and shear-resistance performance of the fracturing fluid was assessed throughout a 25 °C to 200 °C temperature range. For 120 min, the experiments were run with a constant shear rate of 170 s^−1^. A high-pressure sealed concentric cylinder and rotor (PZ38 b) were used by the rheometer, and 32 mL of sample volume was needed for testing.

### 3.5. Shear Recovery Performance

The creation and disintegration of fracturing fluid structures were characterized by shear recovery performance. The shear recovery performance was reflected in the areas between the upward and downward curves. The shear recovery of fracturing fluid was measured using PP35/Ti plate by in the rotary mode of HAAKE MARS 40. The test was split into two phases: the first stage involved changing the shear rate from 10 s^−1^ to 1000 s^−1^, and the second stage involved decreasing the shear rate from 1000 s^−1^ to 10 s^−1^. Following these two stages, an evaluation of the fracturing fluid’s shear recovery behavior was conducted.

### 3.6. Viscoelasticity

The viscoelasticity of the fracturing fluid was measured using a HAAKE MARS40 rheometer. The cone-plate geometry system PP35/Ti (diameter: 35 mm, gal: 1 mm) was used for the measurements. At 25 °C, the viscoelasticity was observed while oscillating shearing was occurring. First, the fracturing fluid’s strain sweeping was examined. The viscoelastic strength in the range of 0.01–10 Hz was then ascertained by performing a frequency scanning of the solution utilizing the strain values of the fracturing fluid in the linear viscoelastic zone.

### 3.7. Sand-Carrying Performance

The fracturing fluid and ceramsite particles were mixed at a 20% sand ratio and stirred for 5 min. The well-mixed solution was transferred to a glass cylinder, which was placed in an oven at a temperature of 90 °C for periodic observation. Photos were taken at different times during the static proppant suspension experiment, and the settling velocity of the ceramsites was calculated.

### 3.8. Gel-Breaking Test

As a gel breaker, a certain amount of ammonium persulfate was added to the fracturing fluid, which was then uniformly mixed and heated to a constant 200 °C for the gel-breaking test. Using a HAAKE MARS40 rheometer, the viscosity of the gel-breaking liquid was tested at 25 °C and 170 s^−1^ per hour to determine whether the fracturing fluid could be degraded efficiently. After centrifuging and filtering the gel breaking solution to produce a residue, it was dried, weighed, and the residue content was computed using Equation (1):

*M*_r_ = *m*_r_/*V*_0_ × 10^3^ (1)

where *M*_r_ is fracturing fluid residue content (mg/L), *m*_r_ is quality of residue (mg), and *V*_0_ is fracturing fluid volume (mL).

### 3.9. Scanning Electron Microscopy

Scanning electron microscopy (SEM, Quanta 450, FEI, Waltham, MA, USA) was used to analyze the microstructure of fracturing fluid aggregation patterns in aqueous solution. Every sample was dried at 25 °C and then placed in liquid nitrogen to freeze at −50 °C. Using an accelerating voltage of 2.0 kV, scanning electron microscopy was used to examine the frozen surfaces of the samples.

## 4. Conclusions

In order to study the impact of the metal–organic crosslinker’s chemical composition on temperature and the shear resistance of the fracturing fluid under high temperature circumstances, four Zr-based crosslinkers and one Al-Zr-based crosslinker were created. The following key conclusions were reached after comparing the Zr-based and Al-Zr-based crosslinkers based on factors such as rheological behavior, sand-carrying capacity, gel-breaking performance, and microstructure. The synergistic effects of aluminum and zirconium ions on the performance of fracturing fluid were also covered.

(1) When compared to zirconium lactic acid crosslinker (crosslinker 1) and the zirconium lactic acid and ethanediamine crosslinker (crosslinker 2), the fracturing fluid made with zirconium lactic acid, ethanediamine, and sorbitol crosslinker (crosslinker 3) offers the slowest viscosity development and highest final viscosity. This good thermal property was caused by a higher crosslinking density from the increase in components amount of the crosslinker, which was attained by creating more crosslinks along the polymer backbone. This early reduction in shear damage was followed by an increase in viscosity under the later high-temperature conditions.

(2) The zirconium sorbitol, lactic acid, and ethanediamine crosslinker (crosslinker 4) was a better choice for polymer ASC compared with the zirconium lactic acid, ethanediamine, and sorbitol crosslinker (crosslinker 3); crosslinker 4 exhibited faster crosslinking rate and higher final viscosity than crosslinker 3, indicating that crosslinker 4 had higher reactivity than crosslinker 3, a higher crosslinker reactivity of crosslinker, more crosslinking sites, and higher strength of fracturing fluid. Therefore, when the crosslinking agent was synthesized, the component with strong chelating ability was preferred to be added, and the component with weak chelating ability was added later.

(3) The aluminum zirconium sorbitol, lactic acid, and ethanediamine crosslinker (crosslinker 5) possessed better properties of temperature and shear resistance, viscoelasticity, shear recovery, and sand carrying than the zirconium sorbitol, lactic acid, and ethanediamine crosslinker (crosslinker 4) due to the synergistic crosslinking effect of aluminum and zirconium ions, resulting in higher viscous nature and denser crosslinked network of Al-Zr-based fracturing fluid.

(4) The gelation mechanism of Al-Zr-based fracturing fluid was that aluminum ion reacted with carboxyl groups from ASC to produce first crosslinked network when the temperature was below 50 °C. Subsequently, as temperatures continued to rise, the carboxyl groups of the ASC crosslinked with primary complexed zirconium ion in situ form second crosslinked network. When temperature increased to over 140 °C, the crosslinking of a secondary complexed zirconium ion produced a third crosslinked network. Tertiary release crosslinking strategy achieved good high-temperature-resistance performance in the form of continuous crosslinking, which avoided the excessive crosslinking dehydration and reduced the loss of viscosity caused by shear damage in the early stage.

## Figures and Tables

**Table 1:** Gel-breaking tests of Zr-based and Al-Zr-based fracturing fluid.

No	Type of Fracturing Fluid	Apparent Viscosity of Gel Breaking Solution (mPa·s)				Residue Content (mg/L)
		0.5 h	1 h	2 h	4 h	
1	Zr-based	30.8	16.5	5.2	3.2	163.6
2	Al-Zr-based	35.5	17.4	5.5	3.5	185.1

**Table 2:** Properties and compositions of five crosslinkers.

Crosslinker	Metal Type	Component Type	Percentage of Each Component/%					
			ZrOCl_2_·8H_2_O	AlCl_3_	LA	ESD	SOR	DI Water
Crosslinker 1	Zr	LA	15	-	57	-	-	28
Crosslinker 2	Zr	LA+ESD	15	-	19	38	-	28
Crosslinker 3	Zr	LA+ESD+SOR	15	-	8	16	33	28
Crosslinker 4	Zr	SOR+LA+ESD	15	-	8	16	33	28
Crosslinker 5	Zr-Al	SOR+LA+ESD	13	2	8	16	33	28

## Data Availability

The data presented in this study are available on request from the corresponding author.
